# Gender and social class inequalities in higher education: intersectional reflections on a workshop experience

**DOI:** 10.3389/fpsyg.2023.1235065

**Published:** 2024-01-10

**Authors:** Daniela Fernandez, Emily Orazzo, Emma Fry, Alice McMain, Michelle K. Ryan, Chuk Yan Wong, Christopher T. Begeny

**Affiliations:** ^1^Department of Psychology, University of Exeter, Exeter, United Kingdom; ^2^Global Institute for Women’s Leadership, Australian National University, Canberra, ACT, Australia; ^3^Faculty of Economics and Business, Organisational Behaviour, University of Groningen, Groningen, Netherlands

**Keywords:** diversity, higher education, inclusion, intersectionality, workshop, gender, social class

## Abstract

Research about the experiences of underrepresented groups in higher education (HE) demonstrates the persistence of challenges, despite policies and institutional strategies to promote inclusion. Diversity and inclusion policies have been part of the HE agenda for several decades, yet most policies and interventions focus on (a) a given, isolated identity experience (e.g., based solely on gender, social class, or ethnicity) rather than more intersectional approaches to identity; and (b) top-down interventions that do not include participants insights in their design. In this paper, we report a case study of a workshop with students at an elite university that drew on an intersectional approach to social identities (IASI), specifically, looking at gender and social class. We explore three key themes: (a) the importance of group processes, (b) the use of visual techniques, and (c) the institutional tensions and the (de)politicisation of social psychology research. Reflecting on this case study we argue that approaches to identity and inclusion in HE can benefit from intersectionality beyond the use of multi and overlapping identity and social group categories. We argue that research in this space is not neutral and needs to acknowledge researchers’ position about (a) inclusion and diversity, (b) perceptions of participants in research, and (c) the motivation and aims of institutions where the research is conducted. Finally, we discuss the theoretical and practical implications of integrating an intersectional approach within social identity research in HE when focusing on underrepresented groups.

## Introduction

Diversity and inclusion (D&I) have been a key mission of higher education (HE) institutions during the past decades. Although universities have historically been associated with exclusion and elitism (Koutsouris et al., 2022), the benefits associated with participating in HE have led governments to promote broader access to universities for all, regardless of an individual’s background. For example, charters like Athena SWAN and Advance HE’s Race Equality identify and address the challenges for the inclusion of students and staff due to their gender and ethnicity, respectively. Research also demonstrates the positive effects of interventions to improve students’ sense of belonging, for example for Black students in the United States (Brady et al., 2020), African American students (Walton and Cohen, 2011), and women in STEM disciplines (Walton et al., 2015).

Although these interventions are important to improve students’ experiences in HE, they focus on singular identities. However, recognition of multiple systems of disadvantage and exclusion is critical (Nichols and Stahl, 2019). Indeed, intersectionality—that is, the understanding of individuals’ experiences of various kinds of discrimination and disadvantages as intersecting, rather than as singular and independent—is a critical framework for the analysis of educational experiences as complex and imbedded in socio-political lives (Kapilashrami, 2021).

Our project aimed to contribute to this corpus to address two important paradoxes in D&I interventions in HE: (a) the lack of focus, from our knowledge, on the intersection of gender and social class; and (b) the focus on top-down and managerial approaches to inclusion (Koutsouris et al., 2022). To this end, we conducted a workshop following an intersectional approach to social identities (from now IASI workshop), with female and male students from underrepresented social class groups at an elite university. The IASI workshop involved students as active actors in the research processes, whilst creating knowledge about issues that they care about and reflecting on their experiences.

The IASI workshop aimed to emphasise an intersectional approach to social identity in educational settings. We designed this method to promote more engagement from students in research activities in universities, especially considering reported student dissatisfaction and cynicism towards studies about their experiences, and the criticisms of the role of researchers as ‘outsiders’ from participants’ experiences (Bridges, 2001). The use of intersectionality as a theoretical framework creates important challenges for social psychology researchers in D&I, in terms of the self-reflection on their own research epistemologies and methodologies, challenging researchers to analyse their respective positionalities in terms of (a) the institutions where research is conducted and funded, and (b) the views about the relationships between the researchers and participants.

We will first provide a summary of how D&I has been understood in HE settings. Then, we will present intersectionality conceptualisations and their uses as theoretical and methodological frameworks. We will also review how these frameworks have been applied to research in HE settings from a social identity approach. Second, we will report a research experience where we aimed to address the paradoxes described above by conducting a workshop from an intersectional approach to social identity (IASI). Finally, we will discuss the implications that the opportunities and challenges that arise from using this method may have on researchers.

## D&I discourses in HE

Diversity and inclusion have been part of HE’s agenda for the last decades. However, how D&I have been approached by HE—from our perspective—has led to different issues. In the United Kingdom, an important policy agenda has been the Widening Participation strategy, which aims to remove access barriers for students from disadvantaged backgrounds (Connell-Smith and Hubble, 2018) and to promote social mobility (Kettley, 2007). However, despite the positive outcomes in terms of increasing the access of students to HE, this strategy has been criticised from a number of different perspectives. For example, there is a lack of clarity on how universities understand concepts such as ‘Widening Access’ and ‘Widening Participation’. As Coyle et al. (2020) describe, even though these ideas tend to be used synonymously, they communicate different perspectives about inclusion. Widening Access tends to focus on the numbers of students from disadvantaged groups and translates to the increase of representation in HE of underrepresented groups, whereas Widening Participation is more likely to include academic and social experiences after access, seeking more representation of students across universities and subjects (Tonks and Farr, 2003).

How universities understand inclusion is also a matter of debate (Koutsouris et al., 2022), and the concept remains, vague, ambiguous and oversimplified (Stentiford and Koutsouris, 2021). Hence, inclusion has become an abstract and universal concept, when in reality it has been associated with multiple significances and values that can be contradictory (Peña et al., 2022). For instance, the association of inclusion with expansion (Marginson, 2016), with a focus on numbers rather than on understanding students’ experiences. Indeed, inclusion has been approached from a managerial and neoliberal perspective (Koutsouris et al., 2022), where participants themselves (e.g., students and lecturers) are the ones that must ensure inclusion in their everyday interactions, rather than tackling systems of exclusion. This lack of problematization can be one of the reasons why diversity initiatives have been shown to be ineffective (Moreu et al., 2021).

Connectedly, interventions to promote inclusion in HE are likely to be designed outside of students’ groups, who often are the intended beneficiaries of such interventions, leading to a notion of participants as ‘receiving’ the intervention rather than co-creating it. This ‘deficit’ perspective of inclusion is built on the idea of educational settings as homogeneous, where one group is recognised as the ‘different’ one (Peña et al., 2022). This notion promotes a fixed idea about what inclusion means, as educational settings are diverse and reunite multiple actors. Instead, a more appropriate definition of inclusion in HE settings considers inclusion as a dynamic and relational process (Peña et al., 2022). This approach emphasises that inclusion is a set of practises within cultural and historical contexts that, to begin with, are diverse, where (a) differences among individuals are expected and valuable; (b) identities are diverse, changing and producing knowledge; and (c) students’ participation produce inclusion, as students see their perspectives integrate into their educational settings and their social and personal well-being is recognised and valued (Peña et al., 2022).

Moreover, most of the interventions drawn on Widening Participation strategies are more likely to focus on only one identity, despite the recognition of the importance of integrating intersectionality in equality, D&I actions in HE (Kapilashrami, 2021). Although some authors advise focusing interventions only on one potential target audience (e.g., Moreu et al., 2021), research in HE has shown that intersectional experiences are important to understand the occurrence of inclusion/exclusion processes. For example, research has looked at the experiences of ethnic minorities female students and giving attention to their challenges but also potentially valuable resources (García Villa and González Y González, 2014), or interrogating HE policies and strategies that lead to the invisibilisation of women of colour (Nichols and Stahl, 2019).

## An intersectional approach to social identities in higher education

There are several reasons why conducting research looking at social identities and intersectionality can offer important insights about HE and its paradoxes when approaching D&I for several reasons. First, intersectional identities acknowledge individuals’ experiences and systems of inequity, providing important insights into educational and organisational practises in HE. Second, HE itself is an institution that has maintained and reproduced segregation (Reay, 2021), thus resulting in an imperative/parallel need to question and address said mechanisms that promote segregation. Students are key actors in HE and their discourses can highlight how these mechanisms operate in their everyday experiences. Hence, when research focuses on students’ experiences, we need to investigate students’ identity experiences and the structural mechanisms that are reproduced within their experiences.

Hurtado (2017) proposed the idea of ‘intersectional identities’ to describe how social identities are positioned in power structures and hierarchies. Therefore, when stigmatised social identities intersect, they become intersectional identities which—under certain conditions—become more salient and are used to enact oppression (Hurtado, 2017). Theoretically, an intersectional approach to social identity aims to contribute a more complex interpretation of identity processes, where identities are not a reflex caused by a stimulus and become salient (Wijeyesinghe and Jones, 2014). Rather, identities coexist, are negotiated, and become more salient and important under particular social contexts. Hence, from this perspective, intersectionality is understood as ‘mutually constituted relations among social identities’ (Shields, 2008, p.301). Therefore, identities—understood as social categories—can be defined and understood only from their relationship with other social categories (for example, gender can be understood only from its relation to social class).

Research in HE settings integrating an intersectional approach to social identity theory has focused mostly on the gendered and racialised experiences of undergraduate students in HE settings, using interviews as methodological techniques (e.g., Liang et al., 2017), and quantitative methods (Charter, 2020). Students’ experiences have also been analysed outside academic settings (e.g., Ireland et al., 2018), to analyse how society and culture impact individuals’ intersectional gender and race experiences. Moreover, research has also demonstrated attempts to build and create new approaches using both frameworks, such as the pedagogy of social justice education (Hahn Tapper, 2013), working for empowering students towards societal transformation.

However, from our knowledge, the intersection of gender and social class from an intersectional approach to social identities has also not been widely explored. This is problematic because social class is a critical dimension in the persistence of inequality in access (Rubin, 2012; Crawford et al., 2016; Ahn and Davis, 2020), and that gender inequalities in HE settings persist in terms of (a) sense of incompatibility with peers, especially in some disciplines (Cheryan et al., 2009; Starr, 2018; Veldman et al., 2021); (b) being more likely to feel like they are imposters (also known as ‘imposter syndrome’), even when they are numerically the majority (Tao and Gloria, 2019): and (d) being more likely to experience sexual harassment, gender bias and sexism from their classmates and instructors (Kuchynka et al., 2018; Begeny et al., 2020; Eaton et al., 2020)—even in fields where women’s representation has substantially grown (Bloodhart et al., 2020; see also Van Veelen and Derks, 2021).

Despite diverse evidence showing the importance of intersectional experiences for students from underrepresented groups, paradoxically, HE diversity, and inclusion strategies persist to focus solely on top-down approaches. Additionally, these strategies have overlooked the role of social class experiences and, moreover, the role of the intersectional experiences in terms of social class and gender.

## Case study: the IASI workshop

### Overview

Researcher have been widely interested in knowing more about why, despite the efforts of HE institutions to promote diversity and inclusion, challenges persist (Moreu et al., 2021). They might be different reasons to explain this phenomenon. In this study, we argue that key aspects for diversity and inclusion, such as (a) gender, social class, and intersectional experiences; (b) participants’ knowledge, perceptions, and needs regarding diversity and inclusion in HE; and (c) the socio-political context where diversity and inclusion strategies are implemented, have been overlooked. Therefore, this workshop study aimed to address the three key paradoxes in research into to D&I in HE: (a) focusing on single identities, not considering the role of the intersection of social class with other identities, such as gender, despite the stratification of HE system in the United Kingdom; (b) following a top-down approach in how interventions are applied; and (c) the constraints faced by D&I researchers at their organisations (such as HE) when organisations focus on individuals’ coping strategies rather than changing the institution to promote inclusion.

To this end, we developed a workshop with students, taking elements from action research (based on Mertler, 2017) and educative workshop (based on Carrasco et al., 2012) methodologies. Educative workshops are influenced by democratic educational workshops and the theme centred interaction approach, providing more depth in terms of how group processes and interaction develop during the workshop (Carrasco et al., 2012). Educational workshops aim to facilitate learning processes considering both theory and praxis, with a focus on the analysis and reflection on pedagogical processes (Betancourt, 1996). To this end, educational workshops are structured following three phases in each session: (a) icebreaker; (b) main activity; and (c) assessment of the session (Carrasco et al., 2012). For the purpose of this study, we developed a workshop considering the following elements of each approach: (a) the focus on participation and partnership with participants from both approaches; (b) the dialectic and emergent process of research from action research; and (c) the three phases described by educational workshops.

Although action research has been used in research about intersectional experiences in HE (e.g., Bailey et al., 2019; Woolf and Wamba, 2019; López et al., 2022), to our knowledge, it has not considered an intersectional approach to social identities, neither included elements from the educative workshop method (Carrasco et al., 2012). Although educational workshops have been part of educational research in HE (e.g., Carrasco et al., 2012), the model proposed in this study aims to emphasise reflexive and critical thinking from the participants, as well as providing a background to analyse the role of researchers during the workshop process.

Hence, in this paper, we aim to (a) report a case study of a workshop intending to address these paradoxes, and (b) analyse some of the opportunities and challenges that this method entails. The university where this project was set was ranked in the top 5 of the least inclusive universities in the United Kingdom (The Sunday Times, 2020). For the workshop, we drew on the ‘intersectional social identities’ framework (Hurtado, 2017). Following this approach, we intended to explore intersections of disadvantages (being working class and being a woman) recognising that these groups allow for both disadvantage and privilege (e.g., being a working class and man). Hence, we understood social identities as being multiple and intersectional (Gaither, 2018), where the self-concept is complex with identities that are simultaneously salient and overlapping, which are constituted by statuses of gender and social class. Therefore, the use of this workshop as a case provided practical information to reflect on the contributions and limitations of this framework in a HE settings.

The workshop followed an intersectional approach to social identities, drawn on action research and educative workshop principles. In this workshop, we considered the importance of (a) knowing about students’ experiences in their own words; (b) recognising students as active agents with knowledge and expertise on their experiences; and (c) developing material that could be raised with the community and university.

The workshop included four sessions across 2 weeks (meeting twice a week). Each session last between 60 and 90 min. The workshop consisted of the creation and implementation of a collaborative guideline to improve the transitional experiences of first-year students from disadvantaged backgrounds during their transition to university in the pandemic context. The collaborative guideline was a booklet shared as a PDF file. Each page of the booklet included information about the university resources that students could access when facing particular challenges at university, and a photography taken by students as part of a photo-discussion activity. These challenges were selected by the participants and included: University well-being support: how to access it, NHS mental health support, Academic Tools, Understanding mitigation, Meeting new people and social support, Discrimination, inclusion, and support. In the case of ‘University well-being support access’, we also included resources outside university available 24/7. Likewise, for Discrimination, inclusion, and support, we included external support.

The guideline was designed to promote skills acquisition to bolster their academic success and that of their peers from similar backgrounds. Hence, the workshop aimed to address the paradoxes that we identified in D&I interventions by (a) promoting the role of peers’ support in the development of collaborative strategies and shared knowledge about how to enhance academic success, (b) increasing their participation in discussions of institutional strategies of Widening Participation, and (c) including a gendered and socio-economic perspective to address the inequalities related to gender and the socioeconomic inequalities associated with their access and ability to obtain a university degree and to enter the workforce. Hence, we aimed to examine the importance of gender and social class, considering how students’ identities are multiple and intersectional, and their experiences have nuances that must be included in Widening Participation strategies. Indeed, we proposed a focus on widening collaboration strategies, that promote students having an active role in the development of these strategies, and that recognise their own knowledge about the challenges that they have faced in achieving academic success.

### Participants

We recruited participants through (a) the target university social media channels, and (b) newsletters shared via email to students from the target university. The call for participant’s poster stated: ‘Work group widening collaboration in gendered educational settings: Are you a first-generation student or identify as a student from low-income household or working class? Are you keen to share your ideas about helping students during the COVID-19 pandemic and contributing to a more equal and inclusive education?’ The poster also included information about the time of meetings and payment. Before starting the workshop, we conducted a ‘Q&A’ 30 min session for students interested that wanted to know more about the project.

Participants were 10 (2 men^2^, 8 women) undergraduate students that identified themselves as (a) first generation (i.e., students whose parents/relatives did not attend HE), (b) low household income, and/or (c) working-class students. We grouped these identities under the umbrella of working-class identity, defining social class in line with previous work as a sense of membership to a particular social class group, shaped by the perception of where an individual stands, relative to others, considering their economic, educational, and social standing (Manstead, 2018). The number of participants after the first session was nine (for details, see Table 1). Participants received payment for their participation in the workshop in line with minimum wage guidelines.

**Table 1:** IASI workshop: participants number, sessions aims, and activities.

Session number	Participants number	Aims	Activities
1	10	To know the group and set guidelines/boundaries/rules for the focus group. To collaboratively analyse photovoice results. To discuss how to approach guidelines (brainstorming). To discuss expectations and assessment of the first session.	Icebreaker: ‘Mingle, mingle’
			Photos group discussion.
			First ideas about the guideline format and topics to be included.
2	9	To evaluate the first session and discuss expectations about the rest of the planning work. To discuss guideline aims and content. To collaboratively, assess the guideline material. To discuss expectations and assessment of the first session.	Icebreaker: One word to describe last session.
			Last meeting wrap-up using an online whiteboard.
			Discussion about the importance of students’ ‘informal’ knowledge to navigate the university.
			Groups discussion about what piece of advice and university resources (e.g., well-being services and how to reach them) should be included for each topic (breakout rooms).
3	9	To present final guideline material. To collaboratively assess the guideline material.	Icebreaker: Sharing one gift on the chat that represents how do you feel today.
			Check-in the final topic to be covered in the guideline.
			In groups, drafting how to convey information for each topic (using online dashboard).
4	9	To assess overall activity and potential impact of sessions. To create strategies to maintain participants’ social network/support.	Participants were presented with reviewed drafts for each topic.
			Discussion about changes to be made/agreements about the content.
			Discussion about project dissemination.
			Closure: voting for best mug and snack.

### Workshop phases

‘The project was conducted during a lockdown period in the COVID-19 pandemic. Therefore, all the activities were conducted online via Microsoft Teams. Each session included a PowerPoint slide as visual support (with key questions and activities; see Supplementary material for an example). The first two sessions focused on identifying opportunities for collaboration with students from low SES backgrounds: (a) the main concerns and barriers that students from low SES have faced during the COVID-19 pandemic, and (b) how to create a resource that could help the transition of first-year students from low SES to the university, considering the importance of gender experiences. For the first session, we utilised a visual methodology using photography, drawn on elements from photo-elicitation (Harper, 2002) and photovoice (Wang and Burris, 1997) methodologies. The third and fourth sessions focused on creating a resource that signposted first-year students to the resources that could help them to navigate university (see Table 1)’.

## Addressing D&I paradoxes through the IASI workshop: opportunities and challenges

In this section, we reflected on the opportunities and challenges that the IASI workshop method entailed. Following the experience of the workshop, as a research team, we reflected on the experience and analysed how this workshop model can be improve and, hence, applied in the future. First, we would describe the opportunities we detected and that can be helpful to researchers working in educational settings, such as the use of group methods and images in research. Next, we will present the challenges recognised in this experience, regarding to the lack of inclusion of intersectional identities discussions during the workshop.

### Opportunities: group methods and intersectional approaches to social identities research

The IASI workshop can be a method to research group dynamics and processes. Collaborative research methods (e.g., action research, participatory action research) developed by disciplines outside social psychology—such as education—present opportunities to challenge social identity assumptions about identity categories, intergroup relations, and the role of social context. A reflection on methodologies in HE research is critical because of its focus on university students, a population constantly used as participants for different studies (Hanel and Vione, 2016), and also a population that has been under several injustices following the changes in the higher education system, such as increase of fees, privatisation, etc. (Odysseos and Pal, 2018). The inclusion of group methodologies—similar to the social justice aspects from the intersectional approach—can provide more complexity and nuances to research. Indeed, although our project was not framed as a participatory action research, students highlighted its participatory aspect, emphasising the ‘collaborative’ aspect of it, in terms of sharing their opinions and collaborating with a group towards a meaningful outcome.

Moreover, conducting the sessions over time with the same group of participants created some intragroup and ingroup-outgroup dynamics that we observed throughout the workshop. For example, students differentiated themselves from other students with more resources, referring to how they did not have access to economic and material resources helpful to navigate university, and emphasising other aspects of their identity in a positive light, such as hard work and cooperation with others, similar to the idea of social creativity (Haslam et al., 2005). The differentiation process was not only with outgroup members but also within the same group. During the photo discussion, students recognised similarities with other members of the groups, identifying the same experiences and settings portrayed in the photos, such as studying from their bedrooms. However, other students recognised that this experience (having your own bedroom to study) was not part of their experiences, and it was considered a privilege. Finally, creating a group of participants who met over time with a sense of shared identity provided a sense of social support within the group, which was demonstrated as participants shared ideas of how to handle difficulties during the pandemic, for example, signposting university support.

### Opportunities: the use of visual techniques in intersectional approaches to social identities research

Even though there is no unique answer in terms of which method is more appropriate in intersectionality research (e.g., qualitative versus quantitative methods, see Else-Quest and Hyde, 2016; Grabe, 2020), a key aspect to consider is the complexity and nuances that intersectionality approaches offer and, hence, the call for diverse methodologies to—at least try to—capture this complexity.

Furthermore, social identity research from an intersectional approach can benefit from techniques from different disciplines, emphasising the interdisciplinary aspect of intersectionality research. For example, our project included the use of visual data. Visual methods enable participants to reflect on their images and discuss aspects that could be difficult to access or explain without the aid of photography. The use of visual data added nuance to the data and created knowledge that could not otherwise be explored with verbal data only (Jenkins and Boudewijn, 2020). In our project, we based the first session activity on the photovoice methodology proposed by Wang and Burris (1997) and photo-elicitation proposed by Harper (2002). Whilst some use photo-elicitation and photovoice as interchangeable concepts (Bugos et al., 2014), we differentiate aspects of them to establish our methodology. Photovoice is a participatory methodology where participants are asked to express their perspectives regarding a particular social or community issue, through the use of photography. Participants not only create photos during the process but also add their interpretations and reflections about those photos collaboratively with others, including a critical perspective that can undercover power relationships of inequality (Freire, 1970) and generate multiple meanings (Peña Ochoa, 2010). Within social psychology research, there has been an increase in the use of photography to better understand students’ experiences (Latz, 2012; Ingrey, 2013; Cornell et al., 2016), and exploring these experiences from an intersectional approach (Jehangir et al., 2022).

Therefore, we take some elements of the photovoice methodology in our work (Wang and Burris, 1997), particularly the participants as the ones taking the photography, its emphasis on participation and group discussion, and the role of photography to represent participants’ realities and critical community issues (Masterson et al., 2018). We adapted the initial photovoice methodology by inviting students to use their mobile devices to take pictures (Yi-Frazier et al., 2015). In our project, students shared photography about ‘being a student during COVID-19 times’, and indeed the use of photography elicited their discussions about these experiences, but also created a sense of shared experiences and community. The use of photography facilitated a positive sense of shared experiences, consistent with the idea that shared social experiences provide a sense of belonging, social support, and trust with others perceived as similar to them (Allen et al., 2021). For example, one participant referred during the discussion of photography of a laptop:

Even though our courses might be different, or like we’re all completely different people but at the moment it’s been reduced down to the same thing, just a laptop screen on your desk, your bed, wherever it is. Like, everything has been completely turned on its head and this is what everyone is now living with depending on your own situation. Everyone is now in the same boat really.

Furthermore, one of the few times that intersectional identities were mentioned was prompted by a photography discussion. A participant referred to how looking at a picture of the campus made her think about her safety, and elicited gender differences regarding perceptions of lockdown and safety on the streets:

I think in terms of, like the impact – gender’s impact of the pandemic, especially kind of given what’s happened, over the weekend and like the murder of the Sarah Everard lady, you’re kind of, I don’t know, I’m more aware of kind of like, if I am on campus, I am alone on campus, if I am on campus. And you have to be kind of social distanced and isolated from people, so if you are in the library, or on campus you are more likely to be alone. (…) also just going out for walks and stuff, as a woman, I would think about is it dark outside? When you’re going out for a walk or not necessarily taking a route that is maybe in a bit of a sketchy area

Although the perceived danger of walking alone during the lockdown was mentioned only by one female participant during the photography activity, this can also be an indicator of the potential that photography has for an intersectional approach to social identity research in educational settings.

### Challenges: (de)politicisation of intersectional research in social psychology

We recognise that the IASI workshop creates methodological, ethical, and political challenges when studying D&I, especially when the researchers work and participate in the same institution as the participants. Intersectionality shares a different paradigm that provides an opportunity to look at structural inequalities and promote action for social justice, to create political and transformative praxis. For some authors, intersectional approaches are grounded in opposition to the positivist perspective and scientific language in research (Grabe, 2020; Buchanan and Wiklund, 2021). On the contrary, intersectionality can offer a perspective of social problems that are understood as a historic, complex, and subjective process (Greenwood, 2008). However, there are concerns about how the ‘intersectionality’ name might be misused by psychology researchers, focusing more on some aspects of this approach (specifically the subjective and identity interactions) than others (the dynamics of power), leaving out the political and transformative aspect of it (Rodriguez et al., 2016). As researchers, we faced the same paradox. For instance, our project focused on strategies to improve academic success, which can be considered an instrumentalist position (Nichols and Stahl, 2019), usually shared by psychology in educational settings. However, success can have different meanings, which are related to students’ identity experiences (Fernández et al., 2023).

Furthermore, during the first session, students emphasised the structural problems faced by them at their university, asking for a change in university policies and support. However, due to institutional guidelines, our project implied a given topic—in this case, highlighting students’ own resources to navigate HE—rather than proposing a collaborative instance to (a) negotiate the topic of the project, and (b) promote institutional changes. Hence, an important challenge faced during this project was to navigate institutional expectations, our expectations as researchers and the students’ expectations. Although we aimed to integrate an intersectional approach to our project, we also recognised the difficulties faced to conduct a political and transformative praxis when we were part of the same institution that students attended in a critical time, such as the beginning of the COVID-19 pandemic: (a) directly or indirectly, being recognised by participants as part of an institution where they do not feel supported; (b) difficulties to provide concrete solutions to structural problems; and (c) negotiate participants and researchers expectations about the outcomes of the activity. Indeed, during the first session, students shared their discomfort with university support during the pandemic. For example, one participant wrote during an online board activity: *‘Departments need to listen and acknowledge students’ voices-*e.g. *petitions about exams, being overworked, extended deadlines rather than ignoring students’ problems given the current circumstances’*. This example is one tension that D&I researchers face outside of this particular case. Hence, these patterns are also seen in D&I research (ers): many researchers do D&I research but are constrained by HE itself because the topic often cannot be negotiated, and the solutions are also kind of directed towards participants’ coping strategies to navigate HE rather than institutional changes.

Therefore, as mentioned earlier, participants perceived the team as ‘outsiders’ from their group despite the efforts to conduct a project from a participatory approach. This notion likely affected participants’ engagement in the workshop, potentially restricting their responses and discussions. The positions of ‘outsider’ and ‘insider’ in participatory research are intricated and not fixed (Losito et al., 1998) and, hence, need to be part of the researcher’s and participants’ analysis in participatory projects.

Although we initiated the sessions with an ice-breaker activity which was facilitated by the undergraduate student from our team, we did not acknowledge our role as part of the institution, and rather, following the time pressure, we moved forward intending to comply with our calendar. Thus, our workshop, although provides strengths to analyse and promote social change, also entails important challenges in fully understanding and challenging existing power dynamics in HE, by way of how much researchers are willing to negotiate their plans and schedule, and their own role as agents from institutions that might promote tensions within participants. If we as researchers do not recognise these tensions in our own position in educational organisations, and create reflexive spaces to think of our research practises—by way of analysing our analyses (Carrasco et al., 2012) and promoting critical self-reflexivity (Grabe, 2020)—we will keep perpetuating a depoliticisation of intersectional and social psychology.

### The challenge of capturing intersectional experiences

Despite the fact that we included the idea of intersectionality in the project invitation and questions to facilitate discussion (e.g., *‘Do you think your gender and social class affected your experiences?’*), and the majority of participants identified themselves as women and from disadvantaged social class groups, students focused their answers on the role of social class. On only two occasions did students mentioned particular challenges in terms of being a woman and being from a low-income background: in terms of safety during lockdown and in terms of gender discrimination. In this case, three students mentioned that students, especially the ones from underrepresented backgrounds, needed information on how to proceed and look for support when experiencing discrimination experiences, with gender being mentioned as one of the key aspects of these experiences.

Hence, despite trying to facilitate the awareness of intersectionality, participants were more likely to discuss their experiences from a particular identity, in this case, their social class. This difficulty has been described in previous research with university students (e.g., Liang et al., 2017). Students’ motivation to discuss their socioeconomic experiences, rather than gender, was part of the findings from this project, and also the challenges that action research entails. Indeed, for action research, research is a dialogic process, where participants’ beliefs and motivations need to be included in the research process. Hence, despite our interest in analysing the intersection of gender and social class experiences, it is important to acknowledge that it is possible that in HE settings social class experiences were more salient.

Previous research has shown the negative consequences of ‘intersectional awareness’, which is individuals’ view of different identities intersecting (Curtin et al., 2015), which could explain why students did not mention intersectional experiences widely. However, we recognise two potential issues that may explain this outcome. First, it is possible that the situational aspects that might make gender experiences more salient were not included properly in the workshop (e.g., creating examples/activities signalling more directly gender experiences in HE settings, including experiences in students’ courses rather than the university as an all). Moreover, we need to consider that the call to participate was initiated with an invitation in terms of social class experiences, rather than gender. At the same time, it is also possible that HE settings make it harder for students to recognise gender inequalities/disadvantages. Universities are institutions where women are often a numerical majority (UNESCO, 2021), giving a sense of equality due to increased participation. However, just increasing the numbers can also lead to a false sense of equality, making it difficult for individuals to recognise inequalities (see Begeny et al., 2020).

## General discussion

In this paper, we aim to report a workshop experience focused on addressing three key paradoxes in D&I strategies in HE: (a) focusing on single identities, not considering the role of the intersection of social class with other identities, such as gender, despite the stratification of HE system in the United Kingdom; (b) following a top-down approach in how interventions are applied; and (c) the constraints faced by D&I researchers at their organisations (such as HE) when organisations focus on individuals’ coping strategies rather than changing the institution to promote inclusion. We argue that, to address these paradoxes, research needs to incorporate methods that position participants as active and critical individuals. Participants’ own sense-making of their experiences and respective interventions should be incorporated into researchers’ theorization and interpretation of their findings. These reflections shed light on the need to consider the complexity of multiple and intersectional identities when D&I strategies are developed. Hence, this experience provided insights into how institutions might be disconnected from students’ needs and demands and the complexities and tensions in the relationship between students and HE institutions. In this paper, one of the problems in defining and applying D & I strategies is that the intended beneficiaries do not usually take part in elaborating or providing inputs regarding the interventions. This is problematic at different levels: first, because the contents and methods used for the interventions might not align with participants’ context, background, and needs. Second, the act of conducting interventions without participants being consulted or asked could signal a message of ‘top-down’ demands and negatively impact the engagement of participants with these strategies.

One example to counteract these issues was the use of visual data. In this experience, projective techniques described by critical social psychology research (e.g., Peña Ochoa, 2010; Carrasco et al., 2012) were helpful to (a) facilitate students’ participation in online settings; (b) convey the idea that meanings are not unique nor individual, and rather are socially constructed by the group; and (c) analyse how these meanings are not necessarily imposed top-down by researchers, which can often be seen among D&I researchers (e.g., starting with a pre-determinate conceptual framework and then looking for these viewpoints in the data). For example, students were asked to describe how they felt, starting the session by sharing a gif in the chat box; or how they perceived the meeting by choosing from a set of memes offered by the facilitators. Visual techniques attempt to address the limitations of conventional research methods and capture the complexities of an ever-changing society (Liebenberg, 2018).

We also recognise the tensions of proposing a research method more in line with participants’ experiences and perceptions yet expecting participants to recognise ‘intersectional experiences’ and naming them as such. This expectation might lead researchers to think of participants’ experiences in terms of identity categories and to suppose that the experiences participants have been expressly due to the social categories (that we as researchers hypothesise), rather than looking at power structures and dynamics that participants may more clearly point or relate to in their experiences.

Hence, to raise awareness of intersectional identities, HE interventions need to recognise and include in their activities the specificity of intersectional groups (e.g., interventions for working class women). This focus can be the first step to create nuanced support for different groups, promoting students’ sense of social support and trust in HE institutions, leading to a co-creation of a collaborative community across different groups. In the future, a more targeted call and workshop design could help to promote awareness about intersectional identities. For instance, future experiences based on the IASI workshop could include participants from one intersectional group (only working-class women) and create activities that explicitly focus on intersectional experiences (e.g., making salient both gender and social class).

Therefore, although we argued that standard research approaches with minoritised groups have limitations, such as the generalisation of results (Smith and Bond, 2022), the level of participation and engagement of the individuals that are part of the research process, and the secondary role of reflection about how psychological theories might participating in the reproduction of inequalities (Parker, 2007), participatory approaches also entail limitations that need to be acknowledged. Participatory approaches also present drawbacks, especially in terms of their practicalities, such as the level of engagement and participation from participants in the project conceptualisation (e.g., Gray et al., 2000), potentially imposing participation (Greenwood et al., 1993), and how participants’ ideas are comprehended by researchers as subjective process intervene in researchers’ objectivity (Ratner, 2002). Therefore, it is crucial to acknowledge the limitations of participatory approaches and consider that the reflection presented in this manuscript cannot be generalised to other experiences. Otherwise, participatory approaches will reproduce what they aim to recognise: the importance of participants’ knowledge and the context where this knowledge is created and negotiate.

## Conclusion

Following the workshop experience, future research in HE settings needs to acknowledge the complexities of educational processes in the current context. Hence, we recommend: (a) to promote co-creation instances with participants, as well as a participative to research, considering participants as key actors of the knowledge produced; (b) to use a wide range of techniques and create context-situated methods to integrate the results from these techniques; and (c) to reflect on the organisational, social and cultural context where research is conducted.

## Data availability statement

The raw data supporting the conclusions of this article will be made available by the authors, without undue reservation.

## Ethics statement

The studies involving humans were approved by CLES Psychology Ethics Committee, University of Exeter. The studies were conducted in accordance with the local legislation and institutional requirements. The participants provided their online written informed consent to participate in this study.

## Author contributions

Material preparation was performed by DF, MR, EO, and AM. Data collection was performed by DF, EO, and AM. Data analysis was performed by DF, MR, EO, and EF. Theoretical and conceptual analysis was performed by DF, MR, EO, EF, CW, and CB. The first draft of the manuscript was written by DF, EO, and EF. All authors contributed to the article and approved the submitted version.
