# Supplementary material for: Gender and social class inequalities in higher education: intersectional reflections on a workshop experience
Source: Front Psychol. 2024 Jan 10;14:1235065. doi: 10.3389/fpsyg.2023.1235065 (PMC10807459; doi:10.3389/fpsyg.2023.1235065)
Supplement: Supplementary file 1 [file Presentation_1.pdf]

Example of workshop’s materials– Session 1

Aims

(a) To meet the final workshop group.

(b) To collaboratively analyse the photovoice results in a safe space.

(c) To discuss guideline format and content.

(d) To discuss expectations and evaluate the first session.

Reminders:

Participation, confidentiality, respect and co/mutual learning.

If you have a prefer pronoun (she/he/they), feel free to let us know.

We invite you to, if you feel comfortable, turn on your camera (this is not mandatory).

Icebreaker activity in breakout rooms:
“Mingle, Mingle”

Group analysis

Describe your/the Picture.

What is Happening in your/the picture?

Why did you take a photo Of this?

What does this photo Tell us about being a student? (Do you think that the photo says something about your experiences as female/male/non binary student? Do you think that the photo says something about how different groups experience being a student at the University?).

How does this photo provide us an Opportunity to learn about your experiences at University during COVID-19 times?

Example of PHOTO discussion.

P
H
O
T
O

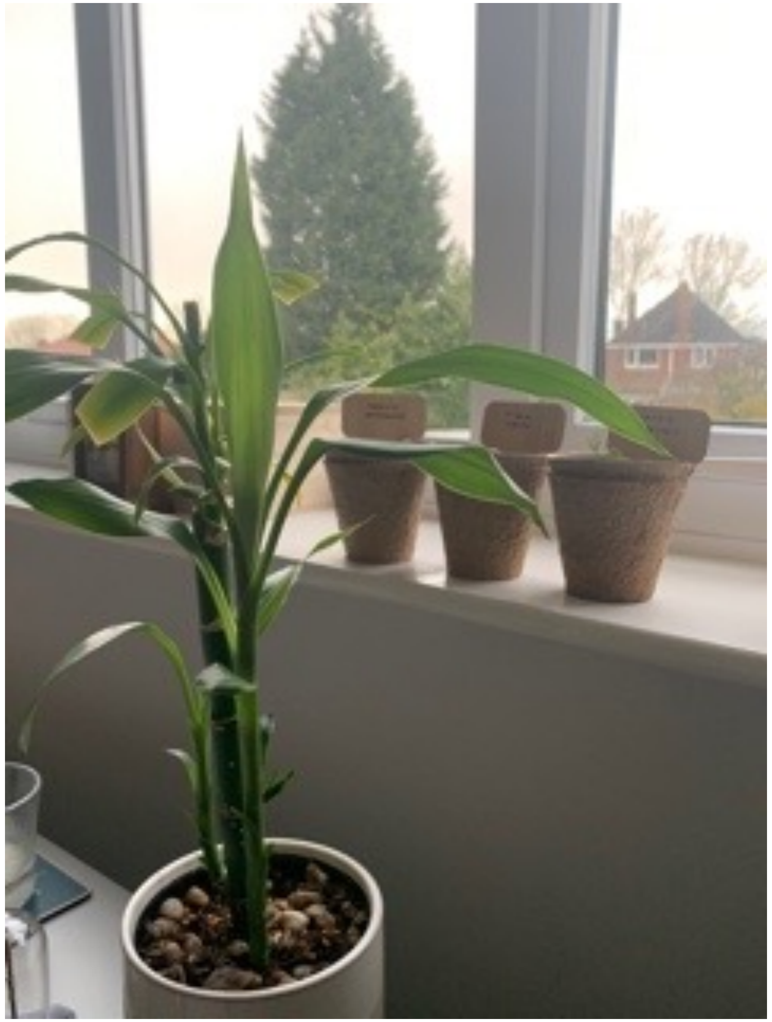

What we can do with the pictures (include them in the guideline material, prepare an online exposition, etc.)?

Where we are?

Where do we want to be?

How we can reach where we want to be?

And how we will share our ideas?
(Guideline format)

Thanks for your participation and all the work that you put into our project!

To start the guideline's writing, we want to ask you to select 2 concepts/themes written on the screen, and develop one page for each about how you will present them on the academic support guideline (definitions, pictures, maps, etc.).

You can use internet resources, books, personal experiences, etc. Participants must send the one page via email to the facilitator to be discussed in the next session.

No worries about the typo, format, etc. We will sort out that later!

Send the document to [researcher email] before Thursday.

Closing

Did you enjoy this meeting? Evaluation activity
(choose your meme)

Thanks for your participation! Any questions, please write to [researcher email].

During today, we will send you a brief survey to asses this meeting.

If any of the contents discussed today made you feel distress and/uncomfortable, remember to contact Wellbeing Centre (TRA).

See you on Thursday ☺
